# Autophagy and Inflammation Regulation in Acute Kidney Injury

**DOI:** 10.3389/fphys.2020.576463

**Published:** 2020-09-25

**Authors:** Li Gong, Qingjun Pan, Nianlan Yang

**Affiliations:** ^1^Experimental Animal Center, Nanfang Hospital, Southern Medical University, Guangzhou, China; ^2^Key Laboratory of Prevention and Management of Chronic Kidney Disease of Zhanjiang City, Institute of Nephrology, Affiliated Hospital of Guangdong Medical University, Zhanjiang, China; ^3^School of Health Professions, University of Alabama at Birmingham, Birmingham, AL, United States

**Keywords:** autophagy, inflammatory, acute kidney injury, immune cells, tubular epithelial cells

## Abstract

Autophagy at an appropriate juncture in the cell cycle exerts protective effects in acute kidney injury (AKI), whereas abnormal autophagy may lead to cell death. Inflammatory response plays a pivotal role in the pathophysiological process of kidney injury and repair during AKI. Several studies have reported an interaction between autophagy and inflammation in the pathogenesis of AKI. This review outlines recent advances in the investigation of the role of autophagy in inflammatory response regulation based on the following aspects. (1) Autophagy inhibits inflammatory responses induced in AKI through the regulation of mTOR and AMPK pathways and the inhibition of inflammasomes activation. (2) Autophagy can also help in the regulation of inflammatory responses through the nuclear factor kappa B pathway, which is beneficial to the recovery of kidney tissues. These studies reviewed here provide better insight into the mechanisms underlying the protective effects of the autophagy-inflammatory pathway. Through this review, we suggest that the autophagy-inflammatory pathway may serve as an alternative target for the treatment of AKI.

## Introduction

Acute kidney injury (AKI), a common clinical syndrome, is often associated with the rapid loss of kidney function and high morbidity and mortality rates. AKI is caused by sepsis, nephrotoxicity, or ischemia-reperfusion injury (IRI). The diversity in the population distribution and outcomes of AKI poses clinical diagnostic and therapeutic challenges to physicians (Takabatake et al., 2014). Regardless of the global attention garnered by this clinical condition, the pathophysiological mechanism underlying AKI remains poorly understood.

Autophagy refers to a cellular homeostatic program for the turnover of cellular organelles and proteins, which involves the encapsulation of the aforementioned objects into vesicles, the formation of autophagosomes, fusion with lysosomes to form autolysosomes, and degradation of the encapsulated contents. The products are either removed from the cells or used to synthesize new proteins or organelles (Mizushima et al., 2011; Mizushima and Komatzu, 2011; Tanida, 2011). Based on these observations, researchers have suggested a link between autophagy and disease. The roles of autophagy in cardiovascular disease, kidney diseases, infection, and immunity among others have been discussed in multiple studies. Using autophagy as a treatment target in these diseases may offer therapeutic benefits (Choi et al., 2013; Mizushima, 2018; Levine and Kroemer, 2019).

Inflammation is an important factor in kidney disorders that occur in AKI (Kinsey et al., 2008). AKI is known to be associated with intrarenal and systemic inflammation. Understanding the cellular and molecular mechanisms underlying inflammation is important for identifying effective treatments to prevent or alleviate AKI (Rabb et al., 2016). Autophagy has been widely regarded as a mediator in inflammatory diseases, including inflammatory bowel disease and various autoimmune diseases. Genetic variations in autophagy-related genes (ATG) such as *atg5* and *ulk1* have been associated with susceptibility to inflammatory disorders (Deretic et al., 2013; Deretic and Levine, 2018). Earlier studies have revealed that certain inflammatory cytokines offer therapeutic potential as treatment targets and that the inhibition of inflammation alleviates kidney damage in AKI. In recent years, many studies have found a complex link between autophagy and inflammation, and the understanding of the processes in autophagy and its inflammatory regulation has emerged as an important part of the study of AKI (Choi and Ryter, 2011). The investigation of the occurrence and regulation of autophagy and renal tissue inflammation in AKI may contribute to the diagnosis, prognosis, and treatment of kidney damage. Renal tubular epithelial cells (RTECs) and the innate and adaptive arms of the immune system are associated with the phases of AKI pathogenesis (initiation, maintenance, and resolution; Choi, 2019). A better understanding of the role of autophagy in the inflammatory response will help design effective anti-inflammatory approaches to AKI. This study reviews the progress in the research on autophagy, its inflammatory regulation, and its role in the inflammatory response in AKI. Each of these components will be discussed in subsequent sections.

## Autophagy: A “Self-Eating” Mechanism

**Figure 1:**
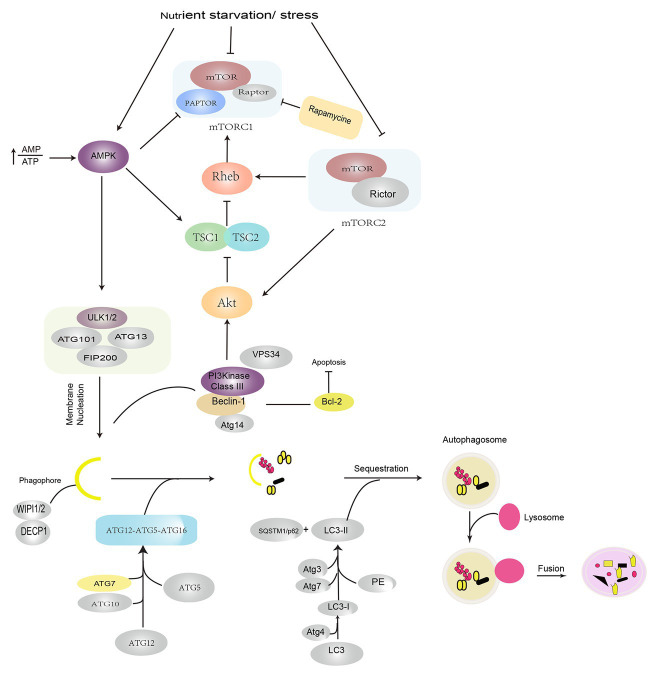
Induction and signaling pathways in autophagy. Autophagy is induced under conditions of nutrient starvation and stress by the activation of AMPK and suppression of the mTOR pathways. The initiation of autophagy is regulated by ULK1/2 complex phosphorylation of the VPS34–Beclin-1 complex, which leads to the recruitment of the cellular PI3P effector proteins DECP1 and WIPI 1/2 and contributes to the formation of an isolated membrane. Autophagosome formation requires the participation of two ubiquitin-like conjugation systems: the ATG12–ATG5–ATG16 complex, which promotes phagophore extension, and LC3/PE, which promotes phagophore closure. After autophagosomes are formed, they fuse with lysosomes and degrade the contents. AMPK: AMP-activated protein kinase; PI3P: phosphatidylinositol-3-phosphate; DECP1: protein 1 containing zinc finger FYVE domain; WIPI 1/2: WD-repeat domain phosphoinositide interaction protein; LC3: light chain 3; VPS34: vacuolar protein sorting 34; PE: phosphatidylethanolamine; ULK1: Unc-51-like kinase 1.

Autophagy is an evolutionarily conserved lysosome-dependent cellular function. Under conditions of intracellular or extracellular stress, cells transport damaged or senescent organelles, or abnormal proteins to lysosomes, which are then degraded upon the mediation of multiple ATG genes and complex cellular signaling pathways (Chen and Klionsky, 2011; Galluzzi et al., 2017), which resultantly helps the cells fulfill their metabolic requirements and enables the renewal of certain organelles, maintenance of the normal cellular structure, and clearance of toxic factors. Depending on the mode of substrate transport, autophagy is divided into three forms: macroautophagy, mediated by bilayer vesicles derived from the endoplasmic reticulum or Golgi bodies; microautophagy, mediated by membrane invaginations, wherein lysosomes directly engulf cytosolic compounds; and chaperone-mediated autophagy (CMA), mediated by selective molecular chaperons. Macroautophagy is commonly referred to as autophagy, which represents the evolution of a series of autophagic structures under strict regulation by various autophagy-related genes under internal and external stresses, such as hypoxia, oxidative stress, starvation, drug stimulation, or the accumulation of intracellular damaged organelles (Hosokawa et al., 2009; Glick et al., 2010). The activation of ULK1/2 (Unc-51-like kinase 1/2) and PI3KC3-C1 (class III-I phosphatidylinositol 3-kinase) is currently thought to be involved in the induction of autophagy. Autophagosome formation is dependent on the vacuolar protein sorting 34 (VPS34)-Beclin-1 complex and the 200-kD focal adhesion kinase family interacting protein (FIP200)-ULK1/ATG1 complex. The elongation and expansion of autophagosomes require two ubiquitin-like proteins: the ATG12-ATG5 conjugate and the microtubule-associated protein 1/light chain 3 (MAP1-LC3/LC3/ATG8). LC3-II formation from LC3 involves the participation of ATG4, ATG7, and ATG3. LC3-II is localized to the autophagosome membrane and is a biological hallmark of autophagy (Hosokawa et al., 2009; Jung et al., 2009; Glick et al., 2010). The serine/threonine protein kinase TOR kinase is a master negative regulator of autophagy. Mammalian target of rapamycin (mTOR) consists of two different complexes, mTORC1 and mTORC2. Specifically, mTORC1 plays a more important role, exhibits greater sensitivity to rapamycin, and is involved in the regulation of growth factors, energy levels, and nutrients; mTOR2 is insensitive to rapamycin and it regulates cell survival and cytoskeleton formation and phosphorylates AKT (Dikic and Elazar, 2018; He et al., 2018). Under nutrient-deficient conditions, dephosphorylation of the mTOR site leads to the dissociation of ULK1, which leads to the phosphorylation of ATG13 and FIP200, and the subsequent induction of autophagy (Choi and Ryter, 2011; Kim et al., 2011; Kim and Guan, 2015). AMP-activated protein kinase (AMPK), which is a key energy sensor, activates autophagy at different steps. First, AMPK directly phosphorylates TSC2, which is the upstream regulator of mTOR, and RAPTOR, a subunit of mTORC1. Under conditions of energy stress, both proteins are phosphorylated and subsequently suppress mTOR activity. The suppression of mTOR activity relieves the inhibitory phosphorylation of ULK1, which leads to the activation of autophagy. In addition to ULK1, AMPK also phosphorylates other core components of the autophagy pathway, such as VPS34, Beclin, and ATG9; however, the molecular details of the regulation of autophagy by AMPK and ULK1 need to be elucidated in the future (Choi and Ryter, 2011; Kim et al., 2011; Kim and Guan, 2015; Sebastien and Reuben, 2018; Figure 1).

## Overview of Autophagy and Inflammation

Autophagy and inflammation are two key physiological and pathological processes. Multiple studies have reported the existence of a complex correlation between autophagy and inflammation (La Paquette et al., 2015; Netea-Maier et al., 2016). Autophagy is essential for the regulation of inflammatory response (Kimura et al., 2017). However, the detailed molecular mechanisms underlying the regulation of inflammatory response by autophagy remain unknown. Pathogen invasion may lead to the generation of pathogen-associated molecular patterns (PAMPs: viral, bacterial, fungal), which further leads to sepsis and subsequently to AKI (Andrade-Oliveira et al., 2015). Invading PAMPs are recognized by plasma membrane-bound toll-like receptors (TLRs). PAMPs may enhance autophagic activation by triggering signaling pathways that involve the participation of mTOR and AMPK. The autophagy inhibitor 3-MA blocks the TLR4/MyD88 pathway and increases the release of inflammatory factors. Studies suggest that autophagy suppresses inflammatory responses in macrophages by regulating the TLR4/MyD88 pathway (Zhao et al., 2019). In a certain study, following stimulation with LPS and TLR4 ligand, Atg16L1-deficient macrophages were observed to produce a significant quantity of inflammatory cytokines IL-1β and IL-18 (Hampe et al., 2007). Similarly, Saitoh et al. (2008) also observed that Atg16L1 was involved in the regulation of LPS-induced activation of inflammasomes in mice. Caspase-1 activation and enhanced IL-1β and IL-18 expression were observed in macrophages in LPS-induced Atg16L1 knockout mice (Saitoh et al., 2008). Colleran et al. demonstrated that autophagy deficiency reduced the severity of injury, possibly owing to the inhibition of the NF-κB pathway. Possibly, the accumulation of ubiquitinated IkB could form a link between the impairment of autophagy and inhibition of NF-κB (Colleran et al., 2011). Nakahira et al. (2011) reported that the depletion of the autophagy proteins LC3B and Beclin-1 increased the secretion of IL-1b and IL-18 and induced the activation of caspase-1 upon LPS stimulation in LC3B-deficient mice.

**Figure 2:**
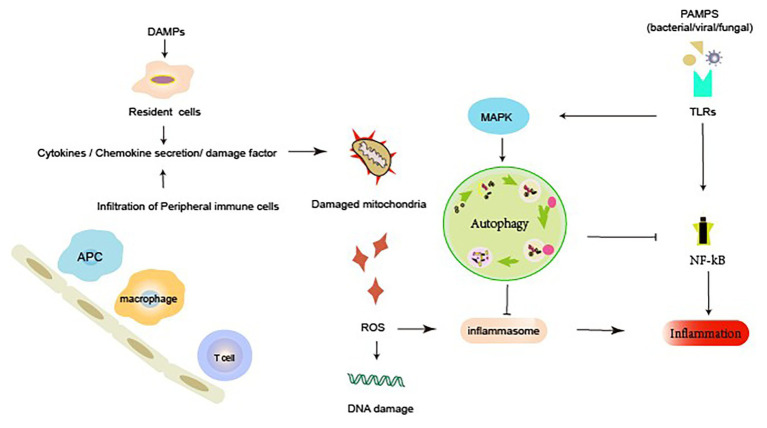
Autophagy and inflammatory response during acute kidney injury (AKI). Renal dysfunction during AKI may result from the external or internal signals involving PRRs, chemokines, cytokines, and ROS. Autophagy can elicit a dynamic response to inflammation. Active autophagy prevents an excessive inflammatory response by preventing the inflammasome activation, mediating the clearance of DAMPs and damaged mitochondria, and degrading inflammatory mediators. DAMP, damage-associated molecular pattern; PAMP, pathogen-associated molecular pattern; PRR, pattern recognition receptors; MAPK, mitogen-activated protein kinase; NF-κB, nuclear factor kappa b; ROS, reactive oxygen species; TLRs, membrane-associated toll-like receptors.

Additionally, sterile inflammation, which occurs frequently in several renal diseases, is triggered by toxins, ischemia, or trauma. This occurs in response to damage-associated molecular patterns (DAMPs) released from injured necrotic cells. DAMPs include histones, DNA/RNA from nucleus, HMGB1, U1snRNP, ATP, mitochondrial DNA (mtDNA), cytosolic RNA, heat shock proteins (HSPs), S100 proteins, uric acid, and lysosomal enzymes from damaged lysosomes. Autophagy also suppresses the release of DAMPs. Endogenous DAMPs are well-known targets for autophagic degradation (Nakahira et al., 2011). Nakahira et al. reported that the depletion of LC3-II or Beclin-1 in autophagy can also promote the dysregulation of mitochondria and substantial accumulation of mitochondrial DNA cytosolic translocation and enhance the activation of caspase-1 and the secretion of IL-1b and IL-18. An earlier study suggested that autophagy proteins contribute to the suppression of inflammatory responses by maintaining mitochondrial integrity (Kim et al., 2011). Autophagy inhibits inflammatory responses through the clearance of DAMPs, such as damaged mitochondria that generate ROS, which can subsequently activate NF-κB signaling and inflammasome formation (Green et al., 2011; Morgan and Liu, 2011). The NLRP3 inflammasome is activated by a broad range of toxic stimuli. Earlier evidence suggests that the NLRP3 inflammasome in macrophages triggers autophagosome formation (Morgan and Liu, 2011). The induction of autophagy depends on the presence of the inflammasome sensor. Autophagy can induce the entrapment and degradation of inflammasomes by inflammasome ubiquitination and the recruitment of the autophagic adapter p62, which facilitates their delivery to autophagosomes. An earlier study suggested that autophagy can moderate inflammation by eliminating active inflammasomes (Shi et al., 2012; Figure 2).

## Inflammation Regulation by Autophagy in Immune Cells in AKI

Almost all immune cells have been implicated in the process of AKI (including initiation, maintenance, and resolution; Bonavia and Singbartl, 2018). Systemic depletion of macrophages in a rat ischemia-reperfusion (I/R) model alleviated renal tubular necrosis, significantly attenuated inflammatory response, and reduced the apoptosis of RETCs by 24 h. Macrophages exhibit two phenotypes in the process of renal injury and repair: proinflammatory M1 phenotype and reparative M2 phenotype (Lee et al., 2011; Huen and Cantley, 2015); proinflammatory M1 phenotype are observed in the first 24–48 h of tissue damage, and the latter occurs in the process of injury resolution (Jo et al., 2006). Dendritic cells (DCs) are present in the renal interstitium and have a protective role in cisplatin-induced AKI (Tadagavadi and Reeves, 2010; Bajwa et al., 2012). It is considered that DCs are the predominant secretors of TNF-α, which serves as a major inflammatory mediator within the first 24 h following IRI. In addition, kidney DCs might promote recovery after I/R injury by phenotypic change from pro-inflammatory to anti-inflammatory (Kim et al., 2010). Depletion of DCs exacerbates cisplatin-induced nephrotoxicity and leads to more severe renal dysfunction, tubular injury, neutrophil infiltration, and mortality in mice (Tadagavadi and Reeves, 2010). In a mouse model of cisplatin-induced AKI, neutrophil infiltration into the kidney and the expression of IL-1β, IL-18, and IL-6 were increased; however, neutrophil depletion and blocking these cytokines did not protect against ischemia injury (Faubel et al., 2007; Bolisetty and Agarwal, 2009).

Although the predominant response to IRI is related to the innate immune system, T cells are very much involved. In the cisplatin-induced AKI model in T cell-deficient mice, it was found that T lymphocytes play a role in experimental cisplatin-induced nephrotoxicity (Burne et al., 2001). Depletion of CD4+ T mice attenuated renal IRI and renal dysfunction (Liu et al., 2006). It is considered that Th1 phenotype (producing IFN-γ) CD4+ T cells are pathogenic, and Th2 phenotype CD4+ T cells are protective. Although the role of CD4+ T cells in ischemic AKI has been confirmed, that of CD8+ T cells in ischemic AKI is still less clear (Yokota et al., 2003). Like macrophages, regulatory T cells (Tregs) in an I/R mouse model play a role in both renal injury and its recovery; depletion of Tregs aggravated renal tubular injury and reduced tubular recovery by increasing T cell proliferation, increasing cytokine levels (Kinsey et al., 2009). NKT cells, which play a bridge between innate and adaptive immunity, are increased by a 3-h return to normal levels by 24 h after I/R injury in mice, and NKT cells participated in the induction of early renal injury by mediating neutrophil IFN-γ production, deficient of NKT cells, significantly alleviating kidney I/R injury in mice (Ascon et al., 2006; Li et al., 2007; Hu et al., 2017). However, in another experimental I/R mouse model, it was found that the loss of NKT cells aggravated the severity of renal injury, while the supplement of NKT cells alleviated kidney injury (Yang et al., 2011). Unlike T cells, which may either promote or repair damage, the role of B cells in ischemic AKI is less known; a limited number of literature showed that the major pathogenic role of B *via* antibodies of the IgM class impaired kidney repair and depletion of B cells in mice models promoted kidney repair (Burne-Taney, 2003; Linfert et al., 2009; Renner et al., 2010).

Autophagy is closely associated with immunity and inflammation. Autophagy contributes to the regulation and function of immune responses (Dong et al., 2007; De Paiva et al., 2009; Monteiro et al., 2009; Singbartl et al., 2018). Macrophages are one of the innate leukocytes that accumulate in the kidney and promote inflammation in the acute phase of AKI (Lech et al., 2014). Zhao et al., (2019) demonstrated that treatment with ursolic acid (UA, a natural pentacyclic triterpene carboxylic acid found in several plants such as apples, bilberries, and cranberries, among others, which promotes cancer cell autophagy) increases autophagy of macrophages and ameliorates LPS-induced AKI. More importantly, treatment with UA enhances macrophage autophagy by increasing the expression of both LC3B and Beclin-1, altering macrophage function, and inhibiting the secretion of inflammatory factors TNF-α, IL-6, and IL-1β in macrophages in response to LPS stimulation. Furthermore, UA inhibits the LPS-induced TLR4/MyD88 pathway. Similar to UA, the autophagy inhibitor 3-MA observably increases the release of inflammatory factors, which indicates the vital role of autophagy in inflammation regulating. A particular study showed that enhancement of macrophage autophagy upon treatment with UA led to the inhibition of the TLR4/MyD88 pathway and other inflammatory processes; these findings indicated that UA might serve as an optimal anti-inflammation therapeutic agent by virtue of its macrophage autophagy-enhancing activity (Zhao et al., 2019). In addition, Andrade-Oliveira et al. (2015) observed that treatment of mice with short-chain fatty acids (SCFAs) improved renal dysfunction caused by injury and reduced oxidative cellular stress, apoptosis, and local and systemic inflammation; they also observed that *TLR4* mRNA and its endogenous ligand, biglycan, when present at low levels, could inhibit NF-κB activation and reduce the infiltration/activation of neutrophils, macrophages, and DCs, and also observed that these factors were associated with an increase in autophagy. However, the study did not reveal whether improved kidney function is associated with increased autophagy in these immune cells itself, neither did it provide direct evidence of the association between immune cell autophagy with inflammatory changes in AKI (Andrade-Oliveira et al., 2015). Chen et al. (2016) observed that the mTOR signaling pathway may serve as a key target for autophagy for the regulation of the inflammatory response by CD4^+^ Foxp3^+^ Tregs after ischemia. The autophagy inducer rapamycin induces autophagy by inhibiting the mTOR signaling pathway, reducing the levels of proinflammatory cytokines (TNF-α, IL-1β, MCP-1, and IFN-γ), and enhancing the expansion of kidney Tregs. The expression of anti-inflammatory cytokines (IL-10 and TGF-β) can be inhibited upon mTOR activation. The adoptive transfer of Tregs with rapamycin treatment can transform kidney macrophages from the M1 phenotype to the favorable M2 phenotype and inhibit the expression of proinflammatory cytokines in kidney CD11b^+^ cells (iNOS, TNF-α, and IL-1β) while enhancing the expression of anti-inflammatory cytokines in kidney CD11b^+^ cells (IL-10, TGF-β). These findings indicate that the enhancement of autophagy in Tregs upon mTOR inhibition significantly inhibits the inflammatory response after ischemic AKI (Chen et al., 2016).

In clinical AKI patients, intrinsic immunologic cells play an important role in kidney injury and inflammatory responses, and autophagy is essential for the regulation of inflammatory responses. These studies have only reported the initial regulation of inflammation by autophagy agonists or inhibitors in AKI. However, there are limited reports of the detailed molecular mechanism underlying the autophagy–inflammatory pathways in immunologic cells in AKI. Therefore, further studies in this area are highly necessary.

## Inflammation Regulation by Autophagy in Tubular Epithelial Cells in AKI

### LPS-Induced AKI

Injury to tubular epithelial cells is a primary feature in AKI. Studies have demonstrated the dysfunction of autophagy and inflammation in tubular epithelial cells during AKI (Mizushima, 2018). Autophagy is associated with LPS-induced inflammatory responses in tubular epithelial cells. In LPS-induced AKI mice, LC3II and Beclin-1 expression and autophagosome formation were enhanced and were accompanied by a concomitant increase in inflammation in the kidney mediated by canonical NF-κB activation. Treatment with 3-MA, which is a pharmacological inhibitor of autophagy, alleviates LPS-induced AKI, inhibits p65 phosphorylation, and results in the accumulation of ubiquitinated IkB (Wu et al., 2015). Leventhal et al. showed that LPS induces RTEC autophagy both *in vivo* and *in vitro* through TLR4-initiated signaling. Proximal tubule-specific knockout of *ATG7* worsens LPS-induced AKI and enhances IL-6 and STAT activation in kidney tissue in response to LPS stimulation. In isolated *Atg7*-KO RTECs, treatment with LPS upregulates IL6 expression and STATS activation (Leventhal et al., 2015). Similar results were also reported in another study, wherein microRNA-30b (miR-30b) was observed to be involved in LPS-induced inflammatory injury and to alleviate autophagy through JNK and NF-κB pathways in HK-2 cells, which belong to an immortalized proximal tubule epithelial cell line derived from normal adult human kidneys. It is commonly known that LPS inhibits HK-2 cell viability and induces the release of inflammatory cytokines (Mizushima, 2018). MicroRNA-30b (miR-30b) inhibits autophagy by activating the JNK and NF-κB signaling pathways and the expression of inflammatory cytokines (TNF-a, IL-1β, and IL-6) and further reduces the viability HK-2 cells upon LPS stimulation, whereas miR-30b silencing exhibits reverse effects. Moreover, the suppression of miR-30b expression alleviates LPS-induced kidney injury in LPS-induced AKI mice by reducing inflammation (Zhang et al., 2018). Zhang et al. investigated the role of ataxia-telangiectasia mutated (ATM) in LPS-induced *in vitro* model of septic AKI and the relationship among ATM expression, tubular epithelial inflammatory response, and autophagy. ATM knockdown in LPS-induced HK-2 cells significantly reduced the levels of LC3 and Beclin-1 and also inhibited the expression of inflammatory factors TNF-α, IL-1β, and IL-6. The study suggested that LPS may induce autophagy in HK-2 cells through the ATM pathway, which could eventually lead to the upregulation of inflammatory factors (Zhang et al., 2019).

### IRI induced AKI

Lipocalin-2 (Lcn2) attenuates renal injury by lowering serum creatinine levels and reducing tubular epithelial cell death in mice. Notably, Lcn2 deficiency reduces autophagy and increases NF-κB activation in renal I/R mice, as Lcn2 protects against renal–ischemia reperfusion injury through autophagy activation mediated by the downregulation of HIF1α and the NF-κB signaling pathway (Qiu et al., 2018). Fibroblast growth factor 10 (FGF10), a multifunctional member of the FGF family, has been reported to exert protective effects against kidney ischemic injury and preserve the histological integrity in a rat model. Activation of autophagy (characterized by upregulation of LC3, Beclin-1, and SQSTM1/p62 expression) and the inhibition of the expression of inflammatory factors (TNF-α, IL-1β, and IL-6) are associated with potential protective activity against AKI. Moreover, rapamycin can partially reverse the renoprotective effect of FGF10, which suggests that the mTOR pathway may be involved in this process (Tan et al., 2018). Apoptosis-stimulating protein two of p53 (ASPP2) is a proapoptotic component of the p53 binding protein family, which plays a key role in regulating apoptosis and cell growth. Compared with wild-type (ASPP2^+/+^) mice, I/R-induced ASPP2 deficiency (ASPP2^+/−^) mice were observed to be protected against renal I/R injury; ASPP2 deficiency was characterized by suppression of inflammation and apoptosis; increased expression of LC3-II, Atg5, Atg7, and Beclin-1; and inhibition of the expression of p62. In addition, the treatment of ASPP2^+/+^ and ASPP2^+/−^ mice with 3-MA or vehicle showed that the protective effect of ASPP2 deficiency in AKI was reversed upon the inhibition of autophagy. These data suggest that downregulation of ASPP2 can ameliorate I/R-induced AKI by activating autophagy and inhibiting inflammation (Ji et al., 2018). Acute zinc chloride treatment in rats exerted a renoprotective effect in ischemic AKI, attenuated endoplasmic reticulum (ER) stress, and inhibited the expression of Beclin-1 and LAMP-2, which was correlated with inhibition of the expression of apoptosis-related factors (caspase-9, caspase-3, and p-JNK) and inflammation-related factors (IL-1ß, IL-6, and MCP-1; Abdallah et al., 2018). C-reactive protein (CRP) was recently reported to be closely associated with poor renal function in patients with AKI. CRP overexpression exacerbated the condition in AKI mice and increased the levels of serum creatinine and urea nitrogen. It was observed that CRP treatment exacerbates oxidative stress *ex vivo* and *in vitro*, and impairs autophagy flux, which is indicated by a low LC-3 II/I ratio and a high level of p62. Rapamycin, which is an autophagy inducer, rescues CRP-impaired autophagy and reduces injury *in vivo*. It has been suggested that CRP worsens kidney ischemic/oxidative injury, which is associated with the downregulation autophagic flux (Bian et al., 2017). Similarly, a young systemic environment effectively alleviated renal I/R injuries in elderly mice, which involved an increase in autophagy and a reduction in oxidative stress and inflammation (Liu et al., 2018).

In addition, 22-oxacalcitriol (OCT), a synthetic vitamin D analog, inhibits the IRI-induced upregulation of TLR4 (IFN-γ) and sodium-hydrogen exchanger-1 (NHE-1) and thereby exerts a renoprotective effect in ischemia AKI by inhibiting autophagy (Hamzawy et al., 2019). Hyperbaric oxygenation (HBO) treatment exerts a renoprotective effect in the IRI model of transplanted rat kidneys, which was observed to be mediated by the activation of cellular autophagy and the inhibition of inflammatory responses (Bao et al., 2017).

### Nephrotoxic AKI

Contrast-induced nephropathy (CIN) is a leading cause of hospital-acquired AKI. The CIN-induced AKI rat model exhibited renal dysfunction, increased mitophagy, mitochondrial fragmentation, ROS generation, and apoptosis in renal tubular cells, alongside increased autophagy and enhanced expression of inflammatory cytokines (IL-6 and TNF-α) in kidneys and serum. The antioxidant 2,3,5,6-tetramethylpyrazine (TMP) can protect cells against CIN. In rats, TMP prevents CIN kidney injury *in vivo* by reversing the associated pathological processes. Mechanistically, TMP efficiently reversed the CM-induced activation of the CCL2/CCR2 pathway, ameliorated renal oxidative stress and aberrant mitochondrial dynamics by restoring the alterations in Drp1 and Mfn2 expression, modulated mitophagy in tubular cells, and suppressed the expression of autophagy genes, including those encoding LC3B, Beclin-1, and p62 protein (Gong et al., 2019). In a cisplatin (a chemotherapeutic drug)-induced model of AKI, treatment of proximal tubule-specific autophagy-deficient mice with cisplatin led to severe mitochondrial damage and promoted ROS production, DNA damage, and p53 activation. Autophagy protects kidney proximal tubules against AKI, possibly by alleviating DNA damage and destroying ROS-generating mitochondria (Takahashi et al., 2012).

**Figure 3:**
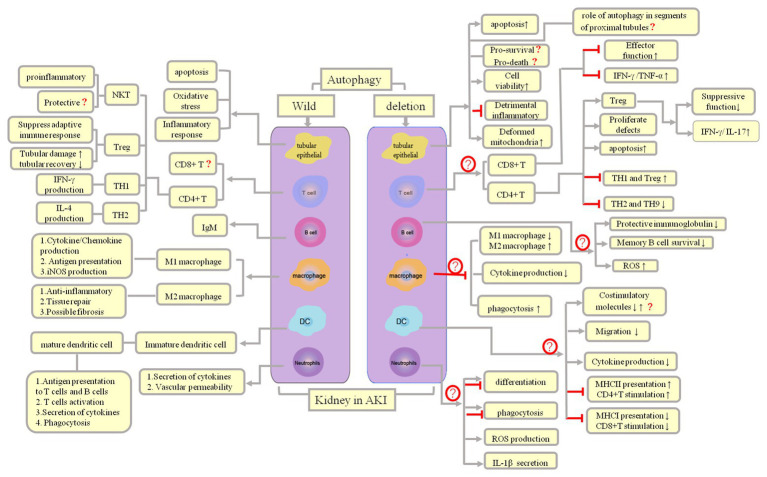
Autophagy and inflammation regulation and outcomes in AKI. Both infectious and noninfectious processes can trigger an inflammatory response, following which parenchymal cells and immune cells can induce AKI, which, in turn, lead to influx of innate immune cells, mainly macrophages, neutrophils, and natural killer (NK) T cells. Both cells of the innate immune system and adaptive immune effector cells are responsible for subsequent damage (Th2 cells plasma B cells) and repair (Th1 and Treg cells, M2 macrophages).

Thus far, studies on the role of autophagy in kidney proximal tubules have often yielded contradictory or unconvincing results (Periyasamy-Thandavan et al., 2008; Inoue et al., 2010; Kimura et al., 2011; Jiang et al., 2012; Liu et al., 2012; Livingston and Dong, 2014; Takabatake et al., 2014; Choi, 2019). We speculate that the reasons for such conflicting data include (1) the use of different autophagy agonists and inhibitors, which are not entirely specific and may have some unknown targets (Kimura et al., 2011; Livingston and Dong, 2014), and when administered at different doses, these autophagy agonists and inhibitors may exhibit different thresholds for autophagy intervention; (2) the differences in the function and degree of damage in different proximal tubule segments (for example, in the AKI mouse model, IRI epithelial cell damage is most apparent in the S3 segment of the proximal tubule, while mitochondrial density is the highest in the S1 segment, and the oxidative burst primarily occurs in the S2 segment; Bao et al., 2017; Gong et al., 2019; therefore, the role of autophagy genes in different proximal tubule segments may differ; one study reported that *Atg5* deletion in the whole proximal tubule exacerbates kidney injury in AKI; Takahashi et al., 2012); and (3) the differences in the sex and strains of the experimental animals. Therefore, it is better to choose the same strain for breeding conditional knockout mice (such as the breeding of Cre/loxp recombinases or the insertion of fluorescent reporter in transgenic mice). The genetic tendencies in offspring of different strains of mice may account for the differences caused by gene deletion. At present, autophagy in tubules and its inflammatory regulation remain poorly understood, and the detailed mechanism needs to be elucidated further (Figure 3).

## Conclusion and Future Directions

Excessive inflammatory responses are a key aspect in AKI pathology. The vital roles played by autophagy in the regulation of kidney inflammation have only been recognized recently. At present, we know that autophagy occurs actively in AKI kidney. Our review suggests that the potential of autophagy to restrict the detrimental effects of inflammation might add to its positive effects in inflammation alleviation (Djavaheri-Mergny et al., 2007). Novel therapeutic interventions designed to enhance autophagy might represent an attractive strategy to overcome insufficiencies in autophagy associated with inflammatory dysregulation during AKI (Duann et al., 2016; Jia et al., 2018; Radi, 2018). Intervention strategies to induce autophagy in various AKI models include the use of autophagy activators, such as rapamycin or its analogs, and autophagy inhibitors, such as chloroquine or 3-MA, both of which have been explored as therapeutic agents in AKI (Djavaheri-Mergny et al., 2007). Additional specific autophagy inducers for potential therapeutic applications include autophagy-inducing peptide (Shi et al., 2012), histone deacetylase inhibitors, and AMPK activators (e.g., metformin and vitamin D analogs). An improved understanding of the complex role of autophagy in inflammation regulation may facilitate the development of potential targets in future therapeutic interventions in AKI.

In addition, the data acquired in rodent models should be critically evaluated in clinically related investigations conducted in the future. The differences in kidney structure and sensitivity to toxic agents (such as LPS and cisplatin) between humans and mice may influence the susceptibility to AKI as well as the degree and nature of inflammation. Moreover, rodents exhibit different results upon AKI injury based on their sex and strains, and we expect differences in the responses between humans and rodents owing to the expression of different immune cell markers and different lymphocyte expression ratios. Autophagy functions may be different in different proximal tubule segments (Milica et al., 2018). Compared to rodents, the kidney structure of a pig is more similar to that of a human. Therefore, studies conducted on a pig model are more conducive to transformation to clinical research, and the humanized mouse model may also partially compensate for such differences. Future studies should focus on the identification of novel AKI animal models that effectively mimic the human inflammatory response, the recognition of novel inflammation mediators and targets for autophagy regulation, and critical assessment of the optimal time points and thresholds for autophagy intervention during AKI. Overall, a precise understanding of the mechanism underlying the regulation of inflammatory response by autophagy will be beneficial in future therapeutic interventions.

## Author Contributions

LG, QP, and NY designed the literature search and wrote the article with input from all authors. LG drafted the manuscript and designed the figures. All authors discussed the results and commented on the manuscript. All authors contributed to the article and approved the submitted version.

### Conflict of Interest

The authors declare that the research was conducted in the absence of any commercial or financial relationships that could be construed as a potential conflict of interest.
