# Corrigendum: Autophagy and Inflammation Regulation in Acute Kidney Injury

**DOI:** 10.3389/fphys.2020.639938

**Published:** 2021-02-09

**Authors:** Li Gong, Qingjun Pan, Nianlan Yang

**Affiliations:** ^1^Experimental Animal Center, Nanfang Hospital, Southern Medical University, Guangzhou, China; ^2^Key Laboratory of Prevention and Management of Chronic Kidney Disease of Zhanjiang City, Institute of Nephrology, Affiliated Hospital of Guangdong Medical University, Zhanjiang, China; ^3^School of Health Professions, University of Alabama at Birmingham, Birmingham, AL, United States

**Keywords:** autophagy, inflammatory, acute kidney injury, immune cells, tubular epithelial cells

An author name was incorrectly spelled as **Qinjun Pan**. The correct spelling is **Qingjun Pan**.

The authors apologize for this error and state that this does not change the scientific conclusions of the article in any way. The original article has been updated.

